# First mass development of *Aedes albopictus* (Diptera: Culicidae)—its surveillance and control in Germany

**DOI:** 10.1007/s00436-016-5356-z

**Published:** 2017-01-23

**Authors:** Norbert Becker, Stefanie Schön, Alexandra-Maria Klein, Ina Ferstl, Ali Kizgin, Egbert Tannich, Carola Kuhn, Björn Pluskota, Artur Jöst

**Affiliations:** 1 Institute for Dipterology (IfD)/KABS, Georg-Peter-Süß-Str. 3, 67346 Speyer, Germany; 2 Faculty of Biosciences, University of Heidelberg, Im Neuenheimer Feld 230, 69120 Heidelberg, Germany; 3 Faculty for Environmental and Natural Resources, Albert-Ludwigs-University, Tennenbacher Str. 4, 79106 Freiburg im Breisgau, Germany; 4 Faculty of Biology, Albert-Ludwigs-University, Hauptstraße 1, 79104 Freiburg im Breisgau, Germany; 5 Bernhard Nocht Institute for Tropical Medicine, Bernhard-Nocht-Str. 74, 20359 Hamburg, Germany; 6 German Centre for Infection Research, Partner site Hamburg-Borstel-Luebeck, Hamburg, Germany; 7 German Environment Agency (UBA), Bötticher Str. 2, 14195 Berlin, Germany

**Keywords:** *Aedes albopictus*, Mass development, Surveillance and control, Germany

## Abstract

The Asian tiger mosquito *Aedes albopictus* has undergone a dramatic expansion of its range in the last few decades. Since its first detection in 2007 in Germany at the motorway A5 coming from Italy via Switzerland to Germany, it has been continuously introduced by vehicles, most probably from Italy. After a hint from an alert gardener in an allotment garden area in Freiburg, Southwest Germany, in 2015, a surveillance programme was started focusing on the garden area and adjacent areas as well as most of the cemeteries as potential infestation areas. The surveillance programme confirmed a high infestation of the allotment garden. The container index (CI) exceeded almost 30% in August 2015. In lethal gravid *Aedes* traps (GATs) and BG-Sentinel traps, 4038 adults were caught. It could be proven that the *Aedes* population is more or less still spatially restricted to the allotment garden area which is adjacent to a train station where trucks from Novara, Italy, arrive loaded on trains. Outside the garden area, only a few breeding sites with developmental stages and adults were found within a radius of approximately 600 m from the highly infested garden area. It is most likely that *Ae. albopictus* females are constantly introduced as ‘blind passengers’ to Freiburg via trucks from Italy to Freiburg, Germany. After the first detection of the mass development of *Ae. albopictus* immediate and comprehensive control measures were initiated to reduce or even eliminate the *Aedes* population. Citizen awareness, especially of the gardeners, was increased by providing thorough information about the biology and control of *Ae. albopictus*. Beside environmental management, tablets based on *Bacillus thuringiensis israelensis (Bti)* were applied. The success of the control activities by the gardeners is reflected by the data gained during monthly inspection of the garden plots. The number of gardens without any container increased from 17% in July to 22% in August and 35% in September, 2015, resulting in a successful reduction of the *Ae. albopictus* population. The study underlines the importance of a comprehensive surveillance programme to assess the population density of *Ae. albopictus* as a basis for integrated control activities.

## Introduction

The Asian tiger mosquito *Aedes albopictus* (Skuse, 1895), originating from Southeast Asia, has undergone a dramatic expansion of its range in the last few decades (Hawley 1988; Cornel and Hunt 1991; Moore and Mitchell 1997; Benedict et al. 2007; Medlock et al. 2012, 2015). The dispersal is facilitated by human activities such as increased mobility of humans as well as international trade, especially the trade with used tyres in which the females lay their eggs (Reiter and Sprenger 1987). The eggs can survive desiccation and dryness for months and thus survive long periods with unfavourable living conditions during transport. Another way of spreading eggs and larvae of *Ae. albopictus* is the trade with ornamental plants (Madon et al. 2004; Scholte and Schaffner 2007).

*Ae. albopictus* possesses a high ecological potency and can rapidly adapt to new habitats due to its genetic plasticity. This species has spread from tropical regions to areas with temperate climates which do not allow a constant follow-up of generations, e.g. during winter periods. As a consequence, the species goes through a winter diapause during which the larvae in the eggs are not able to hatch and remain in the eggshell until the living conditions allow further development. Another important behavioural factor that favours the spread is that *Ae. albopictus* has no strict host preference for blood meals. Adult females predominantly feed on humans but may also bite other mammals including rabbits, dogs, cows and squirrels or occasionally avian hosts. This feeding behaviour indicates that *Ae. albopictus* is well suited for transmitting a variety of arboviruses that use mammals and birds as their main hosts (Mitchell 1995; Gratz 2004).

In Europe, *Ae. albopictus* was first detected at the end of the 1970s in Albania; however, the massive spread started when *Ae. albopictus* was introduced to Italy by used tyres (Adhami and Reiter 1998; Sabatini et al. 1990). Within a few years, the species was established from the north of Italy to Sicily in the south, supported by the wide-scale distribution of used tyres and dispersal by vehicles (Dalla Pozza and Majori 1992; Romi 1994; Roiz et al. 2008). Once established in Italy, the species mainly spread by vehicles, trains or boats to neighbouring countries such as France (French Riviera), Croatia, Spain, Switzerland and across the Alps into Germany (Pluskota et al. 2008; Becker et al. 2012). To date, *Ae. albopictus* has been recorded in 26 European countries (Medlock et al. 2012, 2015).

*Ae. albopictus* is a competent vector of at least 23 arboviruses including Chikungunya, dengue, Zika and yellow fever virus. It has become the most important vector for the Chikungunya virus. In 2007, *Ae. albopictus* was for the first time involved in a small outbreak of Chikungunya fever in the Emilia-Romagna region caused by the tropical virus which was introduced by an infected tourist from India (Pfeffer and Loescher 2006; Reiter et al. 2006; Angelini et al. 2007; Beltrame et al. 2007; Rezza et al. 2007).

As a consequence of this rapid spread of *Ae. albopictus* in the Mediterranean area and the increased public health risk, the German Mosquito Control Association (KABS) started in 2005 a monitoring programme from Basel to Heidelberg along motorway A5 (E35) coming from Italy as a suspected port of entry for *Ae. albopictus* adults. It was assumed that cars, trailers or trucks coming from Italy across the Alps import *Ae. albopictus* to Germany. During the first monitoring programme, along motorway A5 (E35) from Basel to Heidelberg in the period of 2005 to 2009, *Ae. albopictus* eggs were found for the first time in an ovitrap at a resting station north of the city of Weil (Pluskota et al. 2008).

In order to assess the risk for the introduction of *Ae. albopictus*, a collaboration of scientific, traffic and governmental institutions in close cooperation with the public was initiated. The goal is to start control activities immediately when a population of *Ae. albopictus* is identified and to investigate the ports of entry to avoid further introductions (ECDC 2012; EMCA-WHO 2013). In autumn 2014, a private person reported ‘strange’ mosquitoes in an allotment garden area in Freiburg. Accordingly, a local surveillance and control programme was started in 2015 to prove the occurrence and distribution of *Ae. albopictus* as well as to determine the size of the population. The results indicated the first mass development of *Ae. albopictus* in Germany. Here, we report also on the immediate control activities that were conducted to eliminate or at least reduce the population according to a particular action plan.

## Material and methods

### Surveillance

An essential part of the action plan was the surveillance programme to assess the population density, distribution and phenology of *Ae. albopictus*. The surveillance programme has the following goals:

1. Assessment of the way of introduction to prohibit or reduce further introductions
2. Monitoring of the adult *Ae. albopictus* population by employing different trapping techniques
3. Assessment of the container index (CI) at least on a monthly basis

**Fig. 1:**
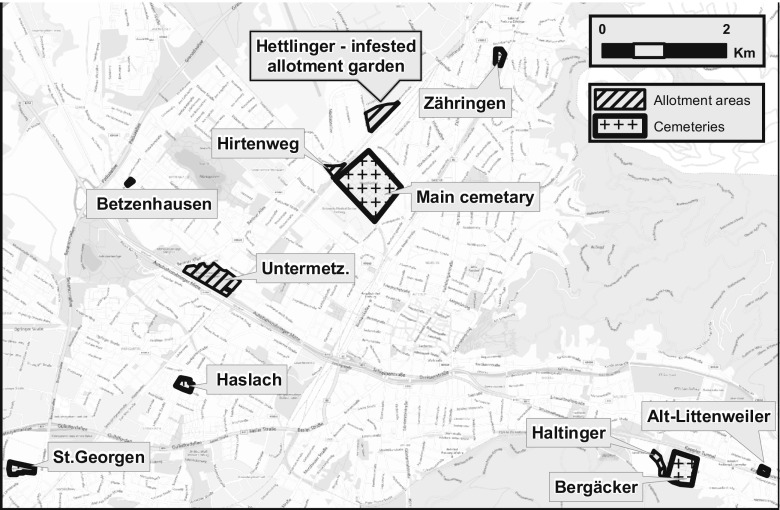
Locations of monitoring in Freiburg

Special attention was paid to the railway station in Freiburg where trucks are reloaded from trains coming from Italy. Trucks are loaded onto trains in Novara in Italy, where *Ae. albopictus* is abundant, and reloaded at the terminal in Freiburg. Approximately 60 trains, each with up to 24 trucks, arrive weekly in Freiburg after passing through the Alps non-stop. It was assumed that *Ae. albopictus* is continuously being introduced by these trucks and escaping from the truck cabins at the terminal to infest the adjacent areas. An allotment garden area (Hettlinger) and a waste disposer including stored used tyres are located close to the terminal, both providing ideal breeding conditions for *Ae. albopictus*. The same is true for the main cemetery of Freiburg which is also located in the vicinity of the terminal. Besides the allotment garden area, the surroundings of the railway terminal including private properties and industrial areas as well as cemeteries were monitored citywide (Fig. 1).

#### Trapping of Aedes albopictus

The following traps were employed in this study to assess the population density and phenology of *Ae. albopictus*:

1. *BG-Sentinel trap.* The BG-Sentinel trap mimics convection of artificial skin emanations which can be found on human skin (ammonia, lactic and caproic acid). The efficacy of the trap was increased by the release of carbon dioxide from a gas bottle and an outlet above the black catch pipe. The release rate was 20 g CO_2_/h. The traps were usually positioned to protect them against wind, rain or direct sunlight (Biogents 2016a).
2. *GAT.* The GAT (gravid *Aedes* trap; Biogents 2016b) is a lethal trap which attracts gravid *Ae. albopictus* female mosquitoes searching for an oviposition place. A black plastic container in the lower part of the trap was filled with hay infusion which attracts females searching for a water body for egg deposition. The hay infusion was prepared by the suspension of 50 g of hay per litre for 48 h. After the female entered the trap through a black funnel, it was trapped inside in a net treated with the pyrethroid alpha-cypermethrin. The trapped female was killed by contact with the insecticide-treated net. Usually, the traps were placed at shaded sites.

#### Set-up of the trapping activities

**Fig. 2:**
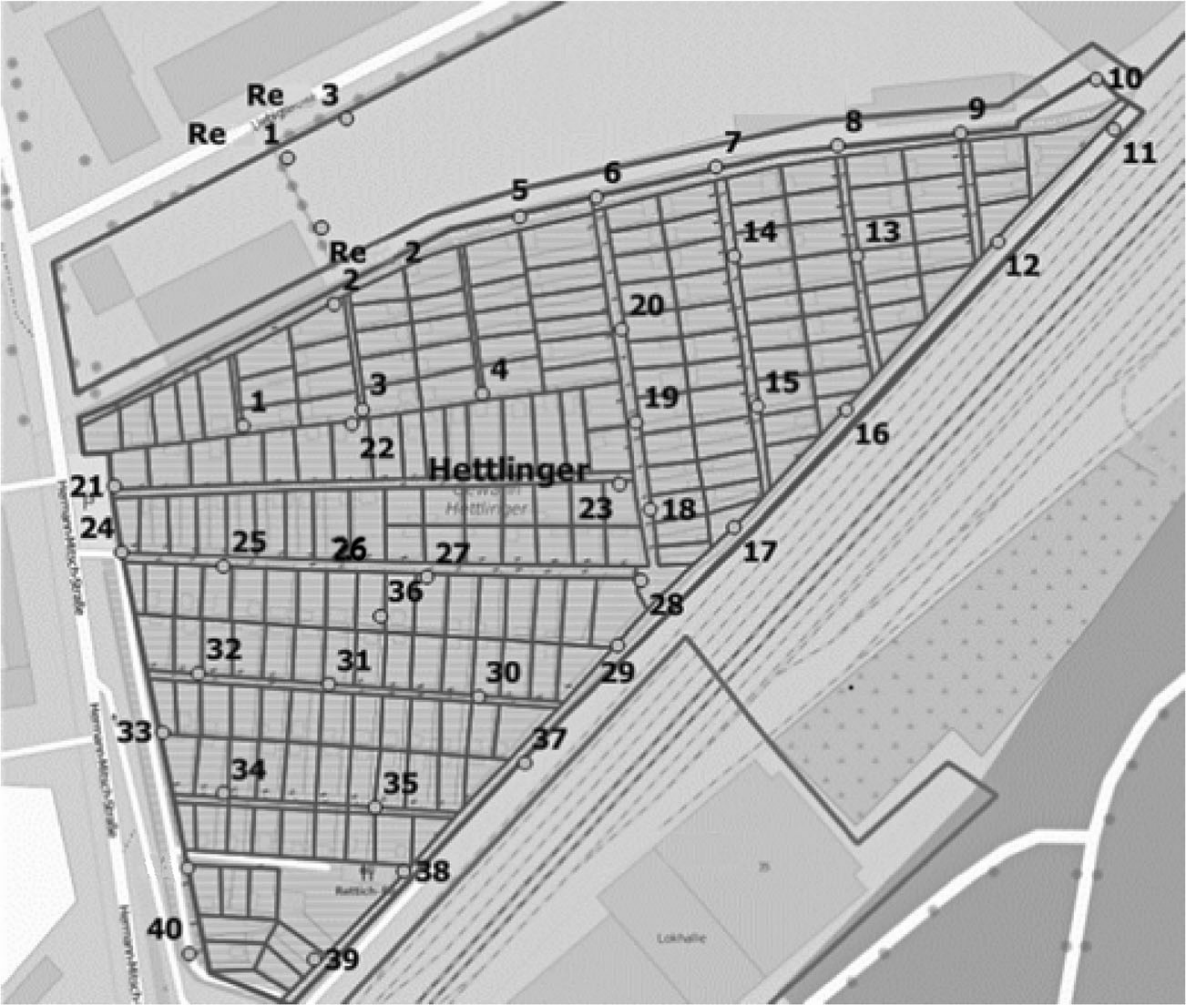
Positions of GATs in the allotment garden area and at the waste disposer

1. *Allotment garden area and adjacent waste disposer.* Two BG-Sentinel traps were placed in the allotment garden area where the main infestation was detected during assessment of the CI. One trap was located at the garden restaurant and one in a garden plot about 90 m from the restaurant. Additional to the 2 BG traps, 40 GATs were placed in transects covering the whole garden area of about 5 ha (Fig. 2). At the adjacent waste disposer, an additional three GATs (Re1-3) were located. The traps were numbered and positioned at shaded locations at a distance of 25–50 m from each other. All traps were checked on a weekly basis; the numbers of captured *Ae. albopictus* were recorded after species determination by morphological characteristics (Becker et al. 2010) or the species was confirmed by PCR sequencing the mitochondrial cytochrome oxidase subunit 1 (CO1) gene when individuals were denudated (Folmer et al. 1994).

2. *Cemeteries.* Two BG-Sentinel traps were placed in the adjacent main cemetery less than 1 km from the allotment garden area. One additional BG-Sentinel trap was located at another cemetery (Bergäcker) about 5 km south-east of the allotment garden area where a small population of *Ae. albopictus* was found (Werner and Kampen 2015). The traps were checked on a weekly basis for *Ae. albopictus*.

#### Sampling of developmental stages

##### Assessment of the container index in the garden area

The infestation of water bodies such as rainwater barrels, buckets, watering cans, flower vases, bird baths or saucers with developmental stages of *Ae. albopictus* was assessed in the whole allotment garden area once a month from July to September 2015. The water bodies were usually inspected by using a torch, and the developing stages were collected with a net for precise determination in the laboratory after Becker et al. (2010). The gardeners were asked to open their garden plots for the inspections. Unfortunately, more than 20% of the garden plots were not accessible due to the absence of the gardeners and locked plots. The presence or absence of developing stages was recorded and the CI was calculated.

##### Extended surveillance of the mass breeding sites citywide with special consideration of the areas surrounding the allotment garden area

Extended surveillance activities in the surroundings of the garden area were carried out to define the spread of the *Ae. albopictus* population. Within a radius of 1000 m from the garden area, four adjacent residential areas and an industrial area as well as the main cemetery of the city of Freiburg were inspected by five groups, each of two people. In private properties, all possible breeding sites, e.g. rainwater containers in garden areas, as well as public water collections, e.g. water catch basins along the streets and grave vases in the cemetery, were monitored for developing stages of *Ae. albopictus*. Detected breeding sites have been controlled with fizzy tablets based on *Bacillus thuringiensis israelensis* (*Bti*) or have been eliminated.

Besides the intensive monitoring of the surroundings of the allotment garden area, a total of seven cemeteries and three garden areas were inspected as expected mass breeding sites. Special attention was drawn to two additional cemeteries due to the records of developmental stages reported in the year 2014 (Werner and Kampen 2015).

### Control of *Ae. albopictus*

Control activities were carried out in close cooperation and thorough flow of information with the federal and local government as well as the city authorities and the health department. The involvement of the public was a crucial element of the programme. The control activities focused on the following goals:

#### Control of Ae. albopictus *by environmental management through community participation*

An essential element of our control operation was environmental management based on community participation. The awareness of control activities by the garden plot owners and inhabitants of the surrounding settlements was increased by the distribution of flyers, fixing of information boards at the entrances of the gardens and cemeteries as well as during several information events. Thorough information on the biology and control of *Ae. albopictus* was provided to give ‘help for self-help’ and to transform the public from ‘spectators’ to ‘actors’ (Becker 1992).

The following control measures were proposed: eliminate unnecessary water bodies; collect, recycle and dispose of unnecessary containers and waste; empty, clean and refill drums, vases, bird baths, etc. on a weekly basis; use waterproof covers for drums, tanks or cisterns; lids or nets have to cover the openings of containers completely to avoid mosquito females laying eggs; storage buckets, water cans and other artificial containers under roofs to avoid the collection of rain water. The implementation of recommendations by the gardeners and house residents was checked by inspections on a monthly basis from July to September.

#### Control of Ae. albopictus *by Bti fizzy tablets*

Water containers which could not be modified by sanitation were treated with Culinex Tab plus (*Bti* fizzy tablets) to eliminate the larvae. The tablets were provided free of charge in the frame of community participation. The 550 mg tablets (Culinex Tab plus, lot no.: D13119, registration no. DE-0003009-18) have a potency of 1000 ITU/mg and contain VectoBac WG (Valent BioSciences, Chicago, USA). The tablets develop a fizzy effect in water and dissolve automatically. The *Bti* fizzy tablets kill only nematoceran flies and do not harm other organisms. Therefore, they are well suited for community participation (Becker et al. 1991; Becker 1992; WHO 1999).

Before operational use, the efficacy of the tablets against *Ae. albopictus* was assessed in bioassays and in semi-field tests.

1. *Assessment of the LC values of the tablets in bioassays against Ae. albopictus.* In the laboratory, bioassays were conducted according to the WHO guidelines for bioassays with some modifications (WHO 1981; Skovmand and Becker 2000). Three *Bti* fizzy tablets were ground to a powder and homogenized; 50 mg of the powder was poured into a 20-mL flask with 10 mL of deionized water and homogenized for 10 min with a vortex. From this initial solution, the basal solution (50 mg/L) was prepared: 0.2 mL of the initial solution was added to 19.8 mL of deionized water and agitated on a vortex at maximum speed. According to the results of a range-finding bioassay, the final concentrations were 0.04, 0.03, 0.02, 0.01 and 0.005 mg/L in 150 mL of deionized water. Four cups were used for each concentration and four cups served as control. In each cup, 25 early fourth instar larvae of *Ae. albopictus* were added. The mortality rate was assessed 24 and 48 h p.a. by counting dead and living larvae. The tests were repeated twice. The mortality data were corrected according to Abbott’s formula (Abbott 1925). The results were subjected to log-probit analysis (Finney 1971). The temperature during the test period was 26 ± 1 °C; the light-dark period was 16 : 8 h.
2. *Assessment of the efficacy of Culinex Tab plus (Bti fizzy tablets) against Ae. albopictus in semi-field tests.* In semi-field tests, rainwater containers (volume: each 220 L) holding 200 L of tap water were positioned in two rows in a shaded garden area in a district close to Heidelberg where *Ae. albopictus* is abundant. Twenty containers were treated with *Bti* fizzy tablets at 2 different dosages. In the first series, ten containers were filled with 200 L of tap water, and in each of five containers, one *Bti* fizzy tablet/100 L or two *Bti* fizzy tablets/100 L (recommended dosage) were randomly applied. In the second series, the same set-up was used; however, in each container, 1 L of water with debris deriving from a natural rain gutter was added to 199 L of tap water. The natural debris was dissolved in 10 L of water. One litre of the suspension was added to each of the containers of the second series. Each of five containers was treated either with one or two *Bti* fizzy tablets/100 L. Three containers served as controls. All containers were covered with tight lids to simulate conditions similar to those barrels completely closed for storing water at homes or allotment gardens. Temperature, pH and conductivity were measured in each container.

Twenty-five third instar larvae of *Ae. albopictus* were released at 3-day intervals into each of the containers. The larvae derived from natural populations in Heidelberg-Wieblingen. Living larvae were removed and counted 72 h p.a. to calculate the mortality. Then, 25 fresh third instar larvae were added to each container. The tests were conducted until the mortality was less than 80% in one of the containers.

The experiment was conducted at a mean ambient temperature of 27.5 °C (18.3–35.9 °C) and a water temperature of 26 °C (18.4–32.9 °C). The pH varied between 6.9 and 7.3 and the conductivity between 785 and 791 μS during the test.

#### Use of GATs to reduce the Ae. albopictus *population*

Besides the use of GATs for monitoring purposes, the efficacy of the lethal GATs to control an *Ae. albopictus* population was assessed in the allotment garden area. The hypothesis was that the *Aedes* population could be significantly reduced by constant elimination of gravid females. Forty traps were numbered and placed in transects covering the whole garden area from July to October 2015 (Fig. 2). The traps were positioned in shaded areas at a distance of 25–50 m. Numbers of captured *Ae. albopictus* were recorded on a weekly basis.

#### Reduction of Aedes *eggs by cleaning the water barrels*

In addition to the control activities during the 2015 season, we aimed to reduce the overwintering larvae in eggshells in spring 2016 before the reproduction period started.

From the beginning of March 2016, a cleaning campaign in the garden area was conducted. All 62 accessible rain barrels in the affected areas used as artificial breeding sites by *Ae. albopictus* were thoroughly cleaned with scrubbing brushes each for about 5 min to remove diapause eggs deposited on the inside of the barrels. The eggs of *Ae. albopictus* are fairly robust and thus can remain intact even after being subjected to the scrubbing process. The debris of each container was filtered through a textile net (white sock) and macroscopically examined by means of binoculars for existing eggs in a whitish plastic pan (20 cm × 20 cm). Then, the plastic trays containing eggs were filled with 1 L of tap water (temperature 25 ± 1 °C) for larval hatching. The hatched larvae from each container were reared to the fourth instar for precise species determination. The effect of the cleaning process was checked during the following time period by inspection of the containers for larval development.

### Statistics

The statistical analysis and graphical documentation was done in SigmaPlot (version 13.0, Systat Software Inc., San Jose, California, USA). The trapping data were tested for normality with a Shapiro–Wilk test and if normality was not given, non-parametric one-way analysis of variance (ANOVA) on ranks by Kruskal–Wallis was performed. Significant differences were approved by Dunn’s test. For the comparison of expected and observed frequencies, a chi-squared test was performed with a threshold value for significant differences of *p* ≤ 0.05.

The mortality rate in the bioassays was corrected according to Abbott’s formula (Abbott 1925). The results were subjected to log-probit analysis (Finney 1971, Raymond 1985), and the data were treated by Duncan’s multiple range test and Student’s *t* test (Köhler et al. 1984).

In the semi-field test, the mortality rates were compared by one-way ANOVA followed by Duncan’s multiple range test.

## Results

### Surveillance

#### Trapping of adults

In the course of the surveillance programme, it was proven that a large population of *Ae. albopictus* developed in the garden area in 2015. A total of 3807 adults were trapped between July and October 2015, namely 847 adults in the two BG traps and 2960 adults in the 40 GATs. Additionally, in the adjacent waste disposer, 231 individuals were caught in 3 GATs (Re 1-3) from August to October 2015.

In comparison to the garden area, only a few individuals of *Ae. albopictus* (five individuals) were caught in the BG traps at the main cemetery. At all other cemeteries within the larger area, no adult *Ae. albopictus* females were detected.

**Fig. 3:**
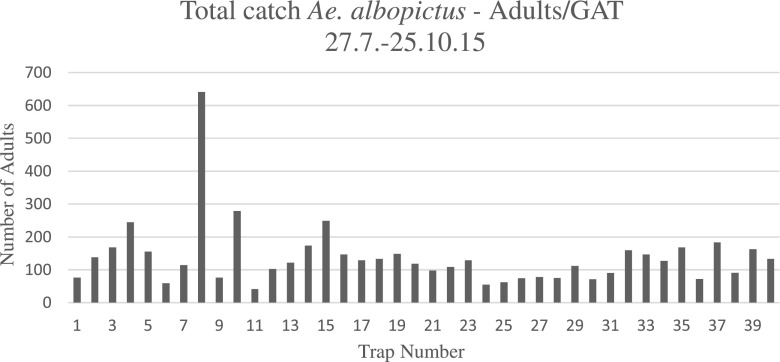
Total number of *Aedes albopictus* adults per trap (nos. 1–40) from the end of July to the end of October 2015

In Fig. 3, the total number of individuals per GAT in the time period from 27th July to 25th October 2015 is presented. The highest number of more than 600 individuals was caught in trap 8, followed by trap 10. Both are close to the used tyre deposit at the waste disposer. Most of the other traps caught between 100 and 200 individuals in 3 months, altogether amounting to 2960 individuals.

#### Container index (CI) at the allotment garden area

**Fig. 4:**
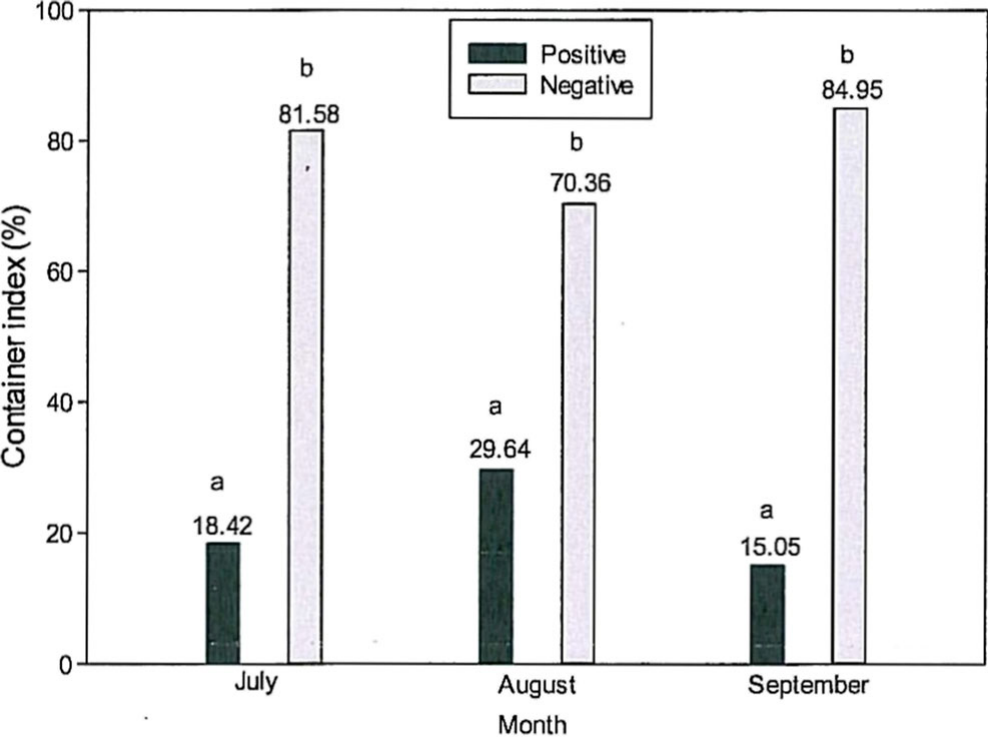
Container indices (CI) (%: positive in *black*, negative in *grey*) in July, August and September 2015. Significant differences (*p* ≤ 0.05) are indicated by different letters (*a*, *b*)

The developing stages of *Ae. albopictus* were sampled in July, August and September 2015. The following spots were examined: in July, 89 garden plots including 190 containers; in August, 151 garden plots including 307 containers and in September, 112 garden plots including 186 containers. The following containers were positive: in July, 35 out of the 190 containers; in August, 91 out of 151 containers and in September, 12 out of the 112 containers, which corresponds to a CI of 18.42% in July, 29.64% in August and 15.05% in September (Fig. 4).

#### Extended surveillance

In the extended surveillance area at nine sites, developing stages and adults of *Ae. albopictus* could be recorded, not more than 600 m from the mass breeding sites. In the residential areas, a water catch basin and two rainwater containers with developing stages of *Ae. albopictus* were identified. In the industrial areas, two adults and at the waste disposer developing stages were found in used tyres as well as biting adults. In the main cemetery, one grave vase was infested with larvae of *Ae. albopictus* and in the two BG-Sentinel traps three adults were caught. In a garden area, one rainwater container with developing stages was recorded.

### Effect of the control activities

#### Reduction of the breeding sites by environmental management through community participation

**Fig. 5:**
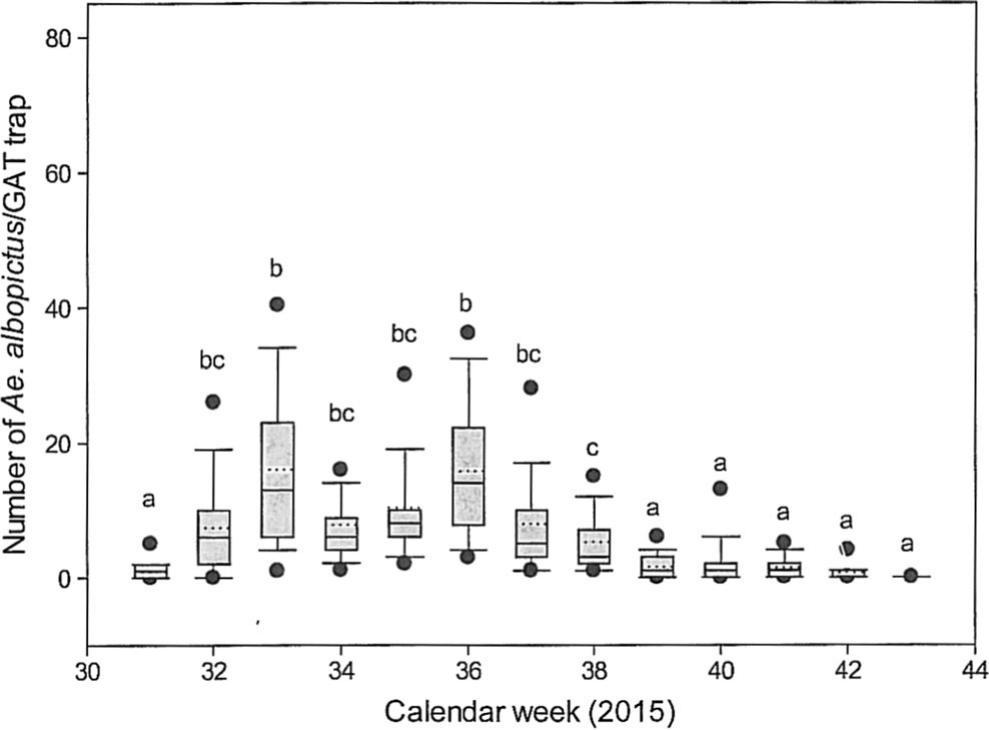
Number of *Ae. albopictus* adults per GAT from week 30 to week 43. Significant differences (*p* ≤ 0.05) are indicated by different letters (*a*, *b* and *c*)

Thorough information of the gardeners resulted in a steady reduction of breeding sites in the garden plots by elimination of water-holding bodies. Inspected gardens without any containers increased from 17% in July to 22% in August and 35% in September. The encouragement of the gardeners to avoid egg laying by gravid *Ae. albopictus* females in rainwater containers by using tight lids, mosquito nets or taping of gaps in the coverage was well accepted. After the maximum abundance of *Ae. albopictus* in the GATs in the 33rd week, the number of *Ae. albopictus* never reached this level afterwards. It is striking that the second peak in the 36th week did not exceed the numbers during the 33rd week, even though the conditions of development were ideal for *Ae. albopictus* (mean temperature week 34–36 19.4 °C; minimum temperature 8.3 °C; precipitation 31.6 mm). From the 36th week, the number of trapped *Ae. albopictus* steadily declined (Fig. 5).

#### Effect of Culinex Tab plus (Bti fizzy tablets)

**Fig. 6:**
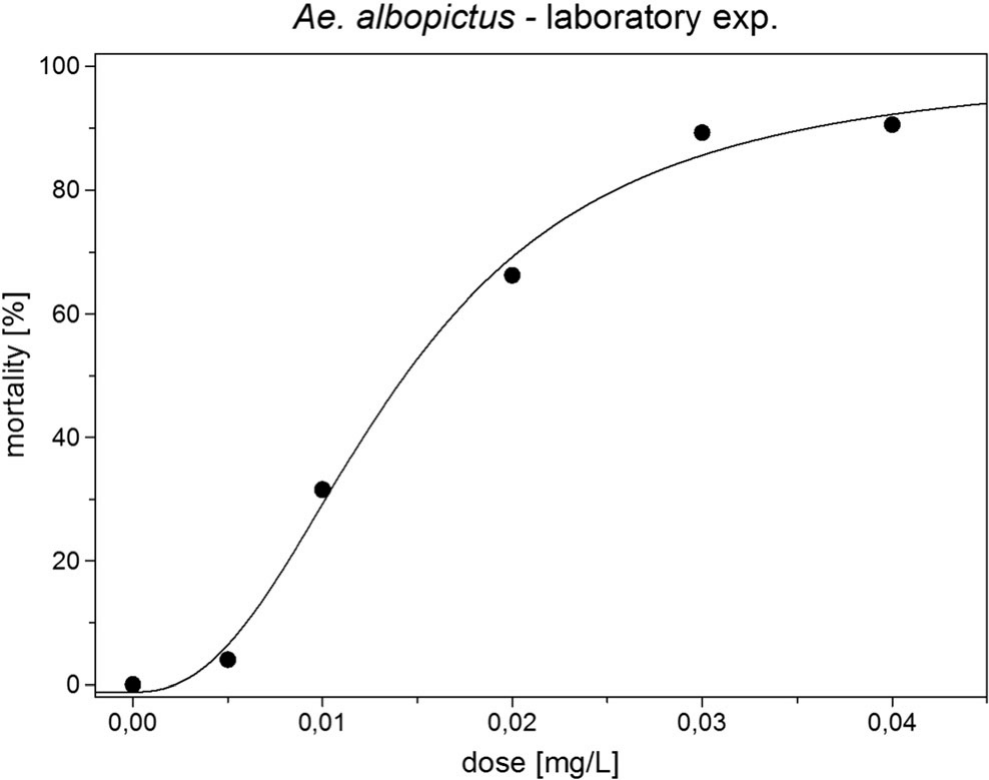
Dose–response curve of early fourth instar larvae of *Aedes albopictus* to Culinex Tab plus (*Bti* fizzy tablets)

1. *Bioassays.* The bioassays with early fourth instar larvae of *Ae. albopictus* revealed an LC_50_ value of 0.0149 mg/L and an LC_90_ value of 0.03558 mg/L (Fig. 6).

2. *Semi-field tests.* In the first series with tap water, the mortality was 100% for 15 days when the recommended dose of one tablet/50 L (11 mg/L) was applied and 94.4% when half of the dosage (5.5 mg/L) was applied. After 24 days, the mortality rates were still 96.8 and 89.6%, respectively.

**Fig. 7:**
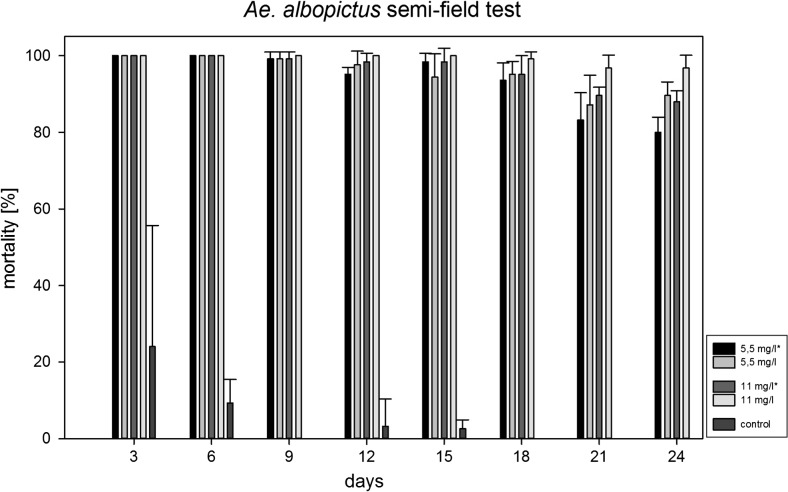
Efficacy of Culinex Tab plus tablets in semi-field tests at dosages of 5.5 mg/L (one tablet/100 L) and 11 mg/L (two tablets/100 L) with and without organic material (*bars* are SD)

In the second series with tap water and organic material from the rain gutter, the mortality was 98.4% at each dosage of one (5.5 mg/L) or two tablets (11 mg/L) after 15 days. After 24 days, the mortality rates were 80 and 88% when one or two tablets/100 L were applied, respectively. The mortality in the control was between 0 and 9.3% between days 6 and 24 (Table 1 and Fig. 7). The slightly higher mortality rate of 24% in the first 3 days was attributed to the acclimatization of the released larvae to the fresh water bodies.

**Table 1:** Efficacy of Culinex Tab plus tablets (%) at dosages of 5.5 mg/L (one tablet/100 L) and 11 mg/L (two tablets/100 L) with and without organic material in semi-field tests

Application	(n) Total	Dead larvae %					
(Days)	(Larvae)	CX^a^ (5.5 mg/L)	CX (5.5 mg/L)	CX^a^ (11 mg/L)	CX (11 mg/L)	CX^a^ (22 mg/L)	Control
0	625	**–**	**–**	**–**	**–**	**–**	**–**
3	625	100 ± 0	100 ± 0	100 ± 0	100 ± 0	100 ± 0	24 ± 31,749
6	625	100 ± 0	100 ± 0	100 ± 0	100 ± 0	100 ± 0	9,3 ± 6,110
9	625	99,2 ± 1,789	99,2 ± 1,789	99,2 ± 1,789	100 ± 0	100 ± 0	0 ± 0
12	675	95,1 ± 1,789	96,6 ± 3,578	98,3 ± 2,191	100 ± 0	100 ± 0	3,2 ± 9,237
15	625	95,1 ± 2,191	94,2 ± 6,066	98,3 ± 3,578	100 ± 0	98 ± 2,828	2,6 ± 2,309
18	625	93,6 ± 4,561	95,2 ± 3,347	95,2 ± 4,382	99,2 ± 1,789	100 ± 0	0 ± 0
21	625	83,2 ± 7,155	87,2 ± 7,694	89,6 ± 2,109	96,8 ± 3,347	96 ± 0	0 ± 0
24	625	80 ± 4	89,6 ± 3,578	88 ± 2,828	96,8 ± 3,346	98 ± 2,828	0 ± 0

^a^Water container loaded with organic material

Duncan’s multiple range test proved high significant differences (*p* < 0.05) between the groups with concentrations of 0.0055 and 0.011 mg/L as well as between groups with and without organic material 21 days p.a.

A total of 167 containers infested with *Ae. albopictus* developmental stages were eliminated or treated with *Bti* tablets. Based on the observations, the use of one tablet per 50 L of water on a bi-weekly basis provided 100% control of larvae.

#### Cleaning of rainwater containers

Out of the 62 scrubbed rainwater containers, eggs could be found in 35 containers (56.45%). All hatched larvae could be determined as *Ae. albopictus*. Larvae could be found in none of the cleaned and re-filled containers in the following 8 weeks of observation in April and May 2016.

## Discussion

This is the first detection of mass development of *Ae. albopictus* in Germany. We define mass development when the CI is >20%; biting adults and several thousand developing stages could be found in a large infested area (>3 ha). Immediate and comprehensive control measures were implemented to reduce or even eliminate the population. The surveillance programme in 2015 confirmed a high infestation of an allotment garden area; however, the *Aedes* population was still more or less spatially restricted to the allotment garden area which is adjacent to a train station where trucks are unloaded from trains arriving from Novara, Italy. Outside the garden area, in the adjacent industrial and residential areas as well as in the main cemetery development stages and a few biting adults were detected only in a few breeding sites such as rainwater containers, water catch basins and vases, but only in a range of not more than approximately 600 m from the allotment garden area. This confirms the limited active spread of *Ae. albopictus* (Becker et al. 2010). It is most likely that *Ae. albopictus* females are constantly introduced as ‘blind passengers’ to Freiburg via trucks. More than 20 trucks can be loaded onto each train in Novara, Italy, an area which is highly infested by *Ae. albopictus* (A. Mosca, personal communication). Each day, more than 100 trucks are transported non-stop by at least 6 trains to Freiburg. It is known that non-blood-fed *Ae. albopictus* females aggressively follow their hosts when they search for a blood meal. Thus, they may enter the driver’s cabin or the accompanying passenger coach in Novara and are released when the cabin is opened after arrival in Freiburg. In the adjacent allotment garden area which is less than 20 m away from the railway station *Ae. albopictus* females find ideal conditions for reproduction, namely a large number of artificial containers such as rainwater containers, water-filled buckets, watering cans or flower pot saucers as well as hosts for blood meals, flowers for sugar meals and bushes as resting zones. Furthermore, the establishment of the species is favoured by the climatic conditions in Freiburg (Pluskota 2011).

The limits for population establishment in Germany are a mean air temperature in January above −3 °C and a mean temperature of the three warmest months (June, July and August) above 19 °C as well as minimum precipitation of 500 mm (Pluskota 2011). In Freiburg, temperature and precipitation measured in the period of 1990 to 2015 were above these thresholds: the January mean air temperature was 2.9 °C, and the mean temperature of the three warmest months (19–20.9 °C) and the annual precipitation at 890 mm exceed the minimum threshold (DWD 2016). The regular introduction of *Ae. albopictus*, the high number of available breeding sites and high summer temperatures for several weeks promoted high population growth within the ‘founder population’. A clear sign of successful establishment was the first demonstration of successful overwintering in Germany in spring 2016 (Pluskota et al. 2016). Most authors assume that the thermal conditions during the overwintering of diapausing embryos in the eggs are the limiting factor (Nawrocki and Hawley 1987; Knudsen 1995; Mitchell 1995; Medlock et al. 2006; ECDC 2009; Takumi et al. 2009). However, studies on the thermal ecology of *Ae. albopictus* revealed that the most important factors for the permanent establishment of the species in Germany are the temperatures occurring during the summer months (Pluskota 2011). In recent years, the average temperatures have risen continually by approximately 1.4 °C in both the summer months as well as in the winter months (Becker 2008). This increases the possibility of successful establishment of *Ae. albopictus* in Germany and makes a continuous surveillance programme and efficient control efforts essential to avoid circumstances as in the Mediterranean area.

Our goal is either to eradicate *Ae. albopictus* or at least to reduce the population to a threshold that does not expose the public to an increased health risk or to reduce the nuisance caused by *Ae. albopictus*. It was important to immediately introduce control activities when the reproduction of *Ae. albopictus* was confirmed. The success of control measures has been documented in the course of a strict monitoring programme. One of the strongest assets for fighting container-breeding mosquitoes is the participation of the community (Becker 1992). Information given to the public about the biology of *Ae. albopictus* and suitable control measures as well as ‘door-to-door’ actions contributed to the decline of the mosquito population. The encouragement of the gardeners to eliminate unnecessary breeding sites, to store buckets or cans under a roof or to turn them upside down, so that no rainwater could be collected as well as modification of the breeding sites such as tight coverage of the containers with mosquito nets or well-fitting lids along with taping small gaps in the coverage was very much accepted. Furthermore, a total of 167 containers infested with *Ae. albopictus* developmental stages were eliminated or successfully treated with *Bti* fizzy tablets. According to the observations, one 550 mg tablet with an activity of 1000 ITU/mg was enough to treat 50 L of water with a killing effect for at least 2 weeks. Similar results were obtained in studies in Indonesia (Becker et al. 1991) and in Columbia by Kroeger et al. (1995). The tablets are safe for the applicant and the *Bti*-treated water can still be used as potable water (WHO 1999).

The success of the control activities by the gardeners is reflected in the data gained by the monthly inspection of the gardens. The number of gardens without any containers increased from 17% (July) to 22% (August) and 35% in September.

However, the CI increased from 18.4% in July to 29.6% in August. The increase of the CI could be partly explained as not all gardeners participated in the control activities and some gardens were not accessible due to the permanent absence of the gardeners and locked garden plots. Furthermore, coverage of the containers was sometimes not appropriate due to gaps in the lids, especially small dark holes which attract females to enter the container because it mimics the situation given by tree holes.

Sensitization and public awareness of control of container-breeding mosquitoes takes time. It is likely that the results obtained in 2016 will be more satisfactory due to early information campaigns. Community participation is the most effective tool but is hard to sustain in the fight against container-breeding mosquitoes. A decrease of the CI can be induced by climatic features like lower temperatures and reduced day length (Roiz et al. 2011). Romi (1995) and Toma et al. (2003) showed that the production of summer eggs ceased when the temperature dropped below 10 °C. The mean temperature in September was 13.26 °C.

The positive effect of the control efforts is well documented in Fig. 5. After the first population peak in the 33rd week, it could be assumed that the second peak in the 36th week would be much higher than the first peak. The population reduction from the 33rd week on was caused by control activities like the elimination and modification of the breeding sites, the reduction of the *Aedes* population by the massive use of GATs and the use of *Bti* fizzy tablets. In total, 4038 *Ae. albopictus* females were caught, especially by GATs (3191 adults). According to Gubler and Bhattacharya (1971) and Hawley (1988), a female lays 300 eggs during its lifespan. Thus, the development of several hundred thousand *Ae. albopictus* offspring in the garden and adjacent waste disposer could be avoided. It could be proven that the lethal GATs can be used as an additional tool to reduce an adult *Ae. albopictus* population. The traps have to be deposited in sufficient numbers at an appropriate distance of about 25 m or less.

The evaluation and control of the *Ae. albopictus* population has continued in 2016 to assess the effect of control efforts based on the results of 2015 and under the consideration of an earlier start of control activities compared to 2015. It turned out that cleaning the containers is a useful additional measure to reduce the starting population of *Ae. albopictus*. None of the 62 rainwater containers which were cleaned by brushing was infested with larvae till May 2016. Thus, the effectiveness of appropriate container cleaning could be proven to combat *Ae. albopictus* in Germany. Similar results have been achieved in Thailand (Phuanukoonnon et al. 2005).

In future years, the sterile insect technique (SIT) and the use of *Wolbachia*-based strategies will be employed to overcome the problem that not all garden plots can be inspected due to the absence or ignorance of the gardeners. We aim to reduce the natural *Ae. albopictus* population by all traditional tools to a level with as few ‘wild’ males as possible survive the control activities. Then, a sufficient number of sterilized or *Wolbachia*-infected males will be released over a sufficiently extended period, so that they outcompete the indigenous males in terms of mating. The males will be either sterilized by gamma radiation (35 Gy) or infected with *Wolbachia*, a bacterial symbiont which leads to cytoplasmic incompatibility. If a female mosquito mates with a sterile or *Wolbachia*-infected male, the female will lay only sterile or semi-sterile eggs and so will not contribute to the next generation of mosquitoes (Seawright et al. 1977; Benedict and Robinson 2003; Bellini 2005; Helinski et al. 2006; Bellini et al. 2007; Brelsfoard and Dobson 2009; Helinski and Knols 2009; Bellini et al. 2013a, 2013b; Balestrino et al. 2014). SIT complements the use of environmental management and products based on *Bti* in an ideal manner because no traditional insecticides are used and the most selective control tools are applied. The defined island-like occurrence of *Ae. albopictus* in Germany in combination with limited migration behaviour favours the use of SIT in an integrated control programme. Furthermore, metallic copper spray could be used to impregnate grave vases as frequent breeding sites of *Ae. albopictus* when a threat to the public exists. Metallic copper sprayed on the interior surface of vases provides a long-term effect and kills newly hatching larvae for up to 3 months (Becker et al. 2015).

Successful control can only be achieved when all effective control tools are carefully implemented and all breeding sites can be accessed. The cooperation of the authorities and the public is essential to be able to inspect all properties.
